# The probability of involvement of human papillomavirus in the carcinogenesis of bladder small cell carcinoma, prostatic ductal adenocarcinoma, and penile squamous cell carcinoma: a case report

**DOI:** 10.1186/1756-0500-7-909

**Published:** 2014-12-15

**Authors:** Soichiro Ogawa, Takahiro Yasui, Kazumi Taguchi, Yukihiro Umemoto, Yoshiyuki Kojima, Kenjiro Kohri

**Affiliations:** Department of Nephro-urology, Nagoya City University Graduate School of Medical Sciences, Nagoya, Japan; Department of Urology, Fukushima Medical University School of Medicine, Fukushima, Japan

**Keywords:** Small cell carcinoma of the bladder, Prostatic ductal adenocarcinoma, Penile squamous cell carcinoma, Human papillomavirus

## Abstract

**Background:**

Human papillomavirus is associated with urogenital carcinogenesis such as penile and uterine cervix cancer. On the other hand, association between human papillomavirus infection and risk of bladder and prostatic cancer remains controversial.

**Case presentation:**

We report a rare case of a 67-year-old Japanese man with synchronous triple urogenital cancer including bladder small cell carcinoma, prostatic ductal adenocarcinoma, and penile squamous cell carcinoma, who presented with a history of asymptomatic gross hematuria. Bladder small cell carcinoma and prostatic ductal adenocarcinoma were diagnosed by histopathological examination after transurethral resection of the tumor. Moreover, he was diagnosed with penile carcinoma based on the exfoliative cytodiagnosis of nodular and papillary tumors inside the preputial collar. He was treated with laparoscopic radical cystectomy, urethrectomy, partial penectomy with pelvic lymph node dissection, and ileal conduit urinary diversion.

To identify a common pathogenesis, we considered human papillomavirus as an etiologic factor because it is a known risk factor for penile carcinoma. Human papillomavirus deoxyribonucleic acid bands were detected by polymerase chain reaction in the three tumors. There was a possibility that human papillomavirus was involved in the carcinogenesis of the triple cancer.

**Conclusions:**

To the best of our knowledge, this is the first report of the synchronous triple urogenital cancer of small cell carcinoma of the bladder, ductal adenocarcinoma of the prostate and penile squamous cell carcinoma. We believe that human papillomavirus may have been involved in the carcinogenesis of the triple urogenital cancer described in this case.

## Background

Small cell carcinoma of the bladder (SmCCB), ductal adenocarcinoma of the prostate (DAP), and penile squamous cell carcinoma (SCC) are relatively rare, each with a morbidity rate of < 1.0 per 100,000 persons per year [1–4]. The etiology of penile cancer is reportedly multifactorial, and human papillomavirus (HPV) is a known risk factor for penile cancer development [4]. However, the causes of SmCCB and DAP have not been elucidated. We report a case of synchronous triple urogenital cancer, including SmCCB, DAP, and penile SCC. Moreover, we discuss the underlying common pathogenesis of the triple cancer.

## Case presentation

A 67-year-old Japanese man presented to the hospital with asymptomatic gross hematuria. He had been smoking for 20 years (20 cigarettes/day). Cystoscopy before transurethral resection (TUR) indicated a nonpapillary sessile tumor on the right bladder wall and a papillary pedunculated tumor on the bladder neck. Histopathological examination of TUR specimens showed muscle-invasive SmCCB and DAP, after which he was referred to our hospital.

**Figure 1:**
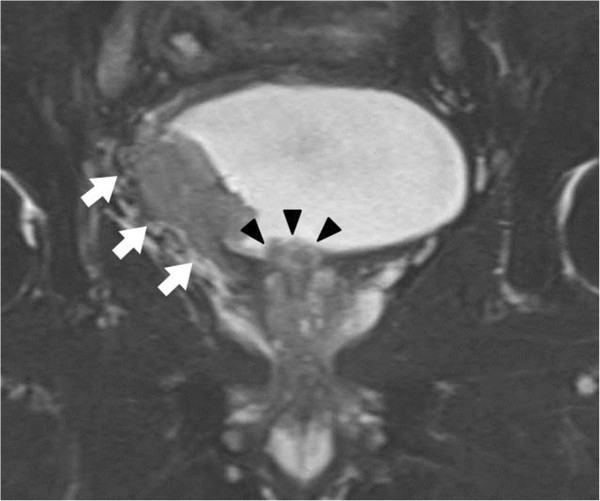
**T2-weighted magnetic resonance imaging showed a locally-invasive tumor (arrows) on the right wall of the bladder and a prostatic urethral tumor that protruded into the bladder (arrowheads), without pelvic lymph node abnormalities.**

Initial physical examination indicated phimosis and nodular and papillary tumors inside the preputial collar. Exfoliative cytodiagnosis of the penile tumor yielded positive results, and suggested SCC. Urinalysis showed hematopyuria, and the urinary cytology results were positive. Serum tumor markers including neuron-specific enolase, pro-gastrin-releasing peptide, and prostate-specific and SCC antigens were within normal limits. Magnetic resonance imaging revealed a locally, muscle-invasive bladder tumor. Moreover, a prostatic urethral tumor protruding into the bladder (Figure 1) and a non-invasive penile tumor were identified, without pelvic lymph node swelling. No remarkable lesions including bone and lung were identified on computed tomography or bone scintigraphy. He was diagnosed with synchronous triple cancer of the bladder, prostate, and penis. He underwent laparoscopic radical cystectomy, urethrectomy, and partial penectomy with an ileal conduit urinary diversion. The obturator and external ilieal lymph nodes were also dissected. No operative complications were noted.

**Figure 2:**
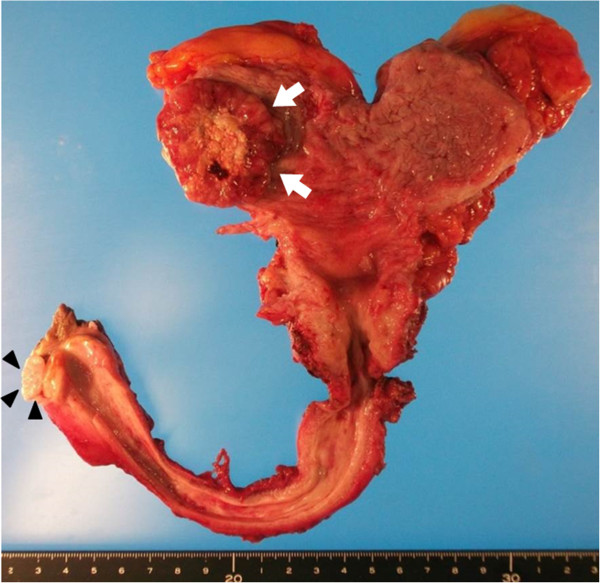
**Macroscopic image of resected en bloc urinary bladder, prostate, urethra, and distal penis.** Arrows indicate bladder tumor and arrowheads indicate penile tumor.

**Figure 3:**
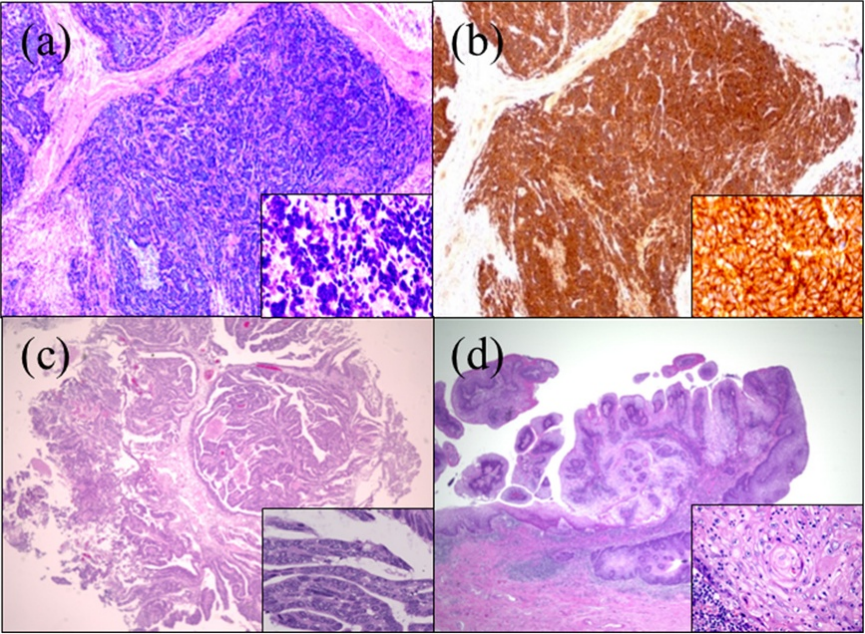
**Histological findings showed triple cancer (magnification of background image: ×40, insert: ×400). (a)** Hematoxylin-eosin (HE) staining of the small cell carcinoma of the bladder. **(b)** Immunohistochemical staining for CD56 demonstrated CD56-positive cells. **(c)** HE staining of the prostatic urethral tumor demonstrated papillary growth and malignancy. **(d)** HE staining of the penis showed cancer pearls and single-cell keratinocytes that are characteristic of squamous cell carcinoma.

The bladder tumor appeared well-circumscribed and solid on gross examination. No tumors were observed in the prostatic urethra. The nodular and papillary penile tumors were located inside the preputial collar (Figure 2). Hematoxylin and eosin (HE) staining of bladder tissue revealed small and round tumor cells closely arranged in cords (Figure 3a). They exhibited positive staining for the neuroendocrine markers synaptophysin (data not shown) and cluster of differentiation (CD 56) (Figure 3b). Therefore, the bladder tumor was diagnosed as a SmCCB (pT3aN0M0). Microscopically, no malignant tumor cells were observed in the prostate. However, HE staining of the TUR specimen was positive for DAP (Figure 3c), which means DAP was completely resected by TUR. HE staining of the penis specimen demonstrated papillary tumor invasion of the submucosal stroma. Furthermore, cancer pearls and single-cell keratinocytes—characteristic of SCC—were found in the penile tumor (Figure 3d), and therefore, it was diagnosed as SCC (pT1N0M0). Thus, he was diagnosed with synchronous triple urogenital cancer, including SmCCB, DAP, and penile SCC.

**Figure 4:**
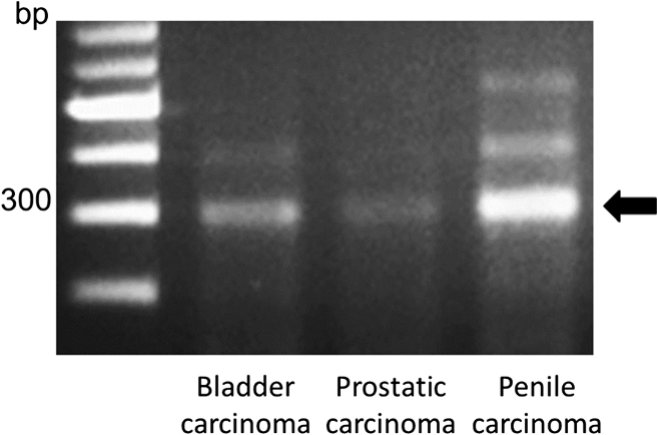
**Human papillomavirus deoxyribonucleic acid was detected by polymerase chain reaction (arrow) in the tumor tissue.**

**Figure 5:**
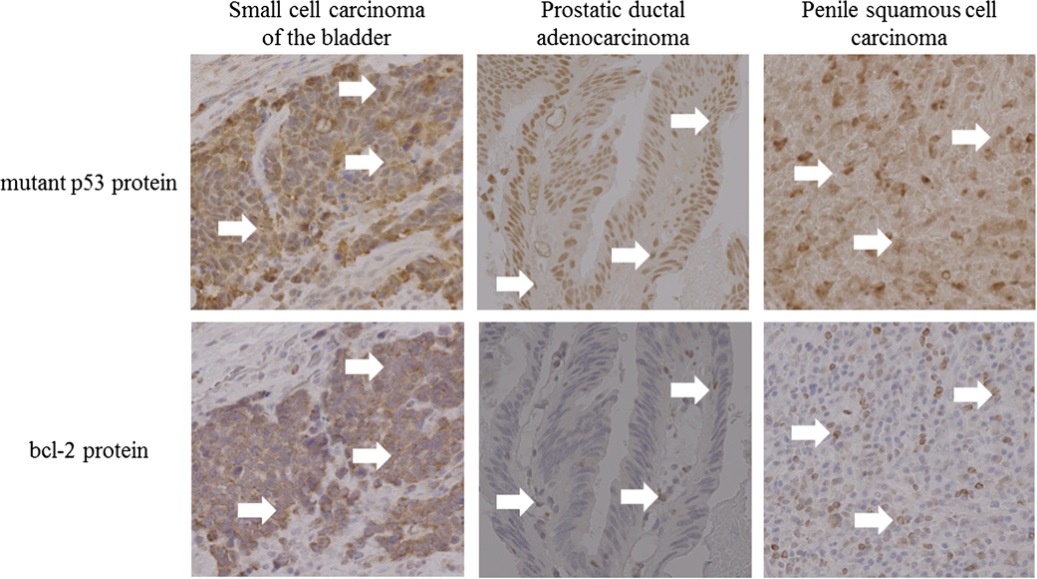
**Immunohistochemical staining showed positive staining for p53 and bcl-2 (arrows) in all three cancers (magnification of background image: ×40).**

To clarify the common pathogenesis, we performed polymerase chain reaction (PCR) tests by using a HPV typing set (6603, Takara Bio Inc). This assay can detect low-risk HPV types, including HPV-6, 11 and high-risk types including HPV-16, 18, 31, 33, 35, 52b. HPV- deoxyribonucleic acid (DNA) containing the domains associated with carcinogenesis (E6 and E7). The assay can amplify PCR products for these domains were detected in all three cancers (Figure 4). Moreover, to examine cell apoptosis, immunohistochemical staining was conducted. Sections were incubated with monoclonal mouse anti-human antibodies for p53 (1:200, Dako) and bcl-2 (1:100, Dako) at 4°C overnight. Next, sections for p53 and bcl-2 were treated with anti-mouse immunoglobulins (Vector Laboratories) for 30 min at room temperature. We obtained positive staining for p53 and bcl-2 in all three cancers (Figure 5).

Since SmCCB was pathologically invasive, the patient underwent adjuvant chemotherapy with cisplatin and irinotecan, based on the treatment regimen for small cell lung cancer. The patient is currently alive at 6 months postoperatively without evidence of tumor recurrence.

## Discussion

SmCCB, DAP, and penile SCC are relatively rare. To identify a common pathogenesis, we considered HPV as an etiologic factor because it is a known risk factor for penile carcinoma. According to their meta-analysis, Li et al. demonstrated a link between bladder cancer and HPV infection based on a significantly increased odds ratio for HPV positivity [5]. Furthermore, recent studies have shown a positive correlation between HPV and prostatic adenocarcinoma risk [6, 7]. It is known that the oncogenic effect of HPV is induced through expression of the oncoproteins E6 and E7 [8]. Therefore, we used the HPV assay kit to identify the variants of HPV associated with the high risk of carcinogenesis. HPV PCR yielded positive results in these tumor cells. HPV serotypes are classified according to low or high oncogenicity into low- or high-risk groups. The latter are known to have a strong oncogenic effect. Thus, our findings suggest that HPV may be the cause of SmCCB, DAP, and penile SCC in this case. However, no positive correlation has been reported between HPV and prostatic adenocarcinoma [9]. Furthermore, Polesel stated that HPV was not involved in the development of bladder cancer [8]. Our results cannot be compared with previous findings because our pathological findings differed from those in previous cases. Further studies such as immunological and genetic basic experiments may clarify whether HPV contributes to the carcinogenesis of DAP and SmCCB.

The E6 oncoprotein complex, synthesized during HPV infection, facilitates the degradation of p53 protein [4, 10] leading to an increased variation of the p53 protein. Consequently, there is an increased expression of bcl-2, which is involved in tumor suppression. Immunohistochemical study demonstrated that variant p53 and bcl-2 proteins were increased in the patient’s bladder, prostate, and penis tumor cells. Thus, we consider the following carcinogenesis mechanism in this case: the degradation of p53 by HPV infection induced cell-cycle dysfunction and caused bcl-2 gene activation, apoptosis suppression, and carcinogenesis.

This study suggests a probability that HPV was linked with carcinogenesis resulting in bladder, prostate, and penile cancers. In addition, it is believed that HPV infected through the urinary tract by the intercourse. However, more detailed information on the association between HPV infection and urogenital cancer is required.

## Conclusions

We described a rare case of synchronous triple urogenital cancer including SmCCB, DAP, and penile SCC. To the best of our knowledge, this is the first report of the synchronous triple urogenital cancer of SmCCB, DAP and penile SCC. We believe that HPV may have been involved in the carcinogenesis of the triple urogenital cancer described in this case.

## Consent

Written informed consent was obtained from the patient for publication of this Case report and any accompanying images. A copy of the written consent is available for review by the Editor of this journal.

## Abbreviations

SmCCB: Small cell carcinoma of the bladder
DAP: Ductal adenocarcinoma of the prostate
SCC: Penile squamous cell carcinoma
HPV: Human papillomavirus
TUR: Transurethral resection
HE: Hematoxylin and eosin
CD: Cluster of differentiation
PCR: Polymerase chain reaction
DNA: Deoxyribonucleic acid
